# The Trabecular Bone Score as a Predictor for Thalassemia-Induced Vertebral Fractures in Northeastern Thailand

**DOI:** 10.1155/2020/4634709

**Published:** 2020-07-21

**Authors:** Nattiya Teawtrakul, Sukanya Chukanhom, Suranut Charoensri, Charoonsak Somboonporn, Chatlert Pongchaiyakul

**Affiliations:** ^1^Division of Hematology, Department of Internal Medicine, Srinagarind Hospital, Faculty of Medicine, Khon Kaen University, Khon Kaen 40002, Thailand; ^2^Division of Hematology, Department of Internal Medicine, Faculty of Medicine, Mahasarakham University, Maha Sarakham 44150, Thailand; ^3^Division of Endocrinology and Metabolism, Department of Internal Medicine, Srinagarind Hospital, Faculty of Medicine, Khon Kaen University, Khon Kaen 40002, Thailand; ^4^Department of Radiology, Srinagarind Hospital, Faculty of Medicine, Khon Kaen University, Khon Kaen 40002, Thailand

## Abstract

**Introduction:**

Thalassemia bone disease is one of the disease-related complications in patients with thalassemia. Prevalence of fractures and the role of a trabecular bone score (TBS) as a predictive factor for fractures were evaluated in patients with thalassemia.

**Methods:**

A cross-sectional study was conducted in patients with thalassemia aged ≥18 years at Srinagarind Hospital, Khon Kaen University, Thailand. A lateral thoracolumbar radiograph and bone mineral density (BMD) at the lumbar spine and hip, as well as the TBS measured by dual-energy X-ray absorptiometry (DXA), were evaluated in all patients.

**Results:**

Among 86 patients, 14 patients were found to have radiographic vertebral fracture yielding a prevalence of 16.3%. All patients who had fractures were *β*-thalassemia/Hb E. Combined low BMD and TBS at lumbar spines and a presence of endocrinopathies were significantly associated with vertebral fractures.

**Conclusions:**

The prevalence of vertebral fractures in patients with thalassemia was not uncommon. A combined low BMD and TBS and a presence of endocrinopathies were associated with vertebral fractures. These findings suggested that BMD testing and TBS measurement have a clinical implication as a screening tool for evaluating the risk of vertebral fractures in thalassemic patients, particularly in *β*-thalassemia/Hb *E* who have endocrinopathies.

## 1. Introduction

Thalassemia syndromes are a group of inherited anemias caused by genetic disorders of the globin genes. The mutations of globin genes have resulted in quantity and quality defects of globin chains. Presently, thalassemia syndromes are classified into 2 main subgroups according to the red blood cell transfusion requirements: (1) Transfusion-Dependent Thalassemia (TDT) and (2) Non-Transfusion-Dependent Thalassemia (NTDT) [1].

Thalassemia bone disease is one of the major disease-related complications in patients with thalassemia. Thalassemia-associated osteoporosis is the most common bone disease in patients with thalassemia. The prevalence varies from 40% to 60% in patients with TDT [2–4]. Evidence has shown that several contributing clinical risk factors are associated with thalassemia-induced osteoporosis including bone marrow expansion [5, 6], iron overload and iron chelation therapy [7–10], endocrine disorders [7, 11–15], vitamin deficiencies [16, 17], and low physical activity [18, 19]. Previous studies reported a wide-range of fracture prevalences in patients with thalassemia (12–51%) [20–22]. A high prevalence of fractures was found in patients with TDT and related to the presence of iron overload-associated endocrinopathy, i.e., hypogonadism, hypothyroidism, and diabetes [20, 21, 23].

The trabecular bone score (TBS) is a new tool to evaluate bone microarchitecture. The score is derived from dual-energy X-ray absorptiometry (DXA) images, measured at the lumbar spine. The TBS was designed to reflect bone quality, while the bone mineral density (BMD) is a proxy for bone quantity. The literature has shown a correlation between the BMD and TBS in the normal population and in patients with thalassemia [24]. The association of low BMD and fractures is well established in patients with thalassemia, but the association between TBS and fractures in these patients remains limited. Therefore, this study was designed to demonstrate the role of TBS as a clinical predictive factor for vertebral fractures in patients with thalassemia.

## 2. Patients and Methods

A cross-sectional study was conducted in patients with thalassemia who were aged 18 years or older at Srinagarind Hospital, Khon Kaen University, between January 2013 and January 2014. Medical history taking and physical examinations were performed by a physician in all patients. Clinical characteristics and laboratory data that the literature indicated as the potential risk factors for thalassemia bone disease and osteoporosis were collected. Endocrinopathies are defined as a presence at least one of the endocrine disorders including (1) diabetes mellitus, (2) hypothyroidism, and (3) hypogonadism. The diagnosis of endocrinopathies was based on medical history, physical examinations, and blood tests. Transfusion-dependent thalassemia (TDT) was defined as a group of patients requiring transfusion of red blood cells every 2–4 weeks to survive. Non-transfusion-dependent thalassemia (NTDT) was characterized as a group of patients who received occasional transfusion of red blood cells.

Symptomatic and radiographic vertebral fractures were recognized. The radiographic vertebral fractures were evaluated by using lateral thoracic-lumbar X-ray radiographs, and the BMDs at the lumbar spine and hip were measured by using the DXA (Lunar prodigy model, GE Lunar). In this study, low BMD was defined as a Z-score from the DXA scanning of less than −2.0 SD. The TBS was derived from the evaluation of the experimental variogram, obtained from the grayscale DXA scan (Medimaps TBS iNsight) at the lumbar spine [25], and low TBS was defined as a TBS value less than 1.1 according to the previous study in patients with thalassemia [24]. The research protocol was approved by the Human Research Ethics Board of the Faculty of Medicine, Khon Kaen University.

### 2.1. Statistical Analyses

Continuous parameters were reported as mean and standard deviations (SD). Categorical parameters were reported as numbers and percentages. Univariate and multivariate logistic regression analyses were used to determine the associations between clinical factors and vertebral fractures. All statistical analyses were performed by the STATA program version 10 (StataCorp, College Station, TX). Statistical significance was considered as a *p* value < 0.05.

## 3. Results

A total of 86 patients (52 females, 34 males) were enrolled. The radiographic vertebral fractures were found in 14 patients (16.3%). Baseline clinical characteristics are shown in Table 1. The mean age was 32.2 years. The mean lumbar spine BMD Z-score was 0.7, while the mean TBS was 1.2. A history of splenectomy was found in 39 patients (45.4%). Nearly half of the patients had a low BMD (36 patients, 42%), and low TBS was found in 7 patients (8%). None of the patients who had a low TBS had a normal BMD. All of the patients who had vertebral fractures were *ß*-thalassemia/Hb E. There were no fractures in patients with *a*-thalassemia.

Univariate analyses of the clinical predictive factors for vertebral fractures in patients with thalassemia are shown in Table 2. Low BMD (odds ratio = 4.4, 95%CI: 1.2–15.5, *p*=0.02), low TBS (odds ratio = 9.2, 95%CI: 1.7–47, *p*=0.008), combined low BMD and TBS (odds ratio = 14, 95%CI: 2.2–86.5, *p*=0.005), a history of splenectomy (odds ratio = 5.7, 95%CI: 1.5–22.5, *p*=0.01), and endocrinopathies (odds ratio = 5.3, 95%CI: 1.5–18.3, *p*=0.007) were significantly associated with vertebral fractures.

**Figure 1:**
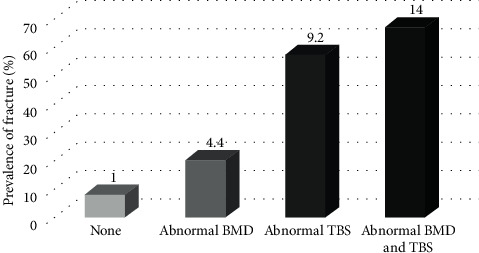
Prevalence of vertebral fractures predicted by the BMD and TBS. The values at the top of the bars are odds ratios (OR).

The patients with combined low BMD and TBS had the highest prevalence of pathological fractures (67%, odds ratio = 14), followed by the patients with low TBS (57%, odds ratio = 9.2) and the patients with low BMD (20%, odds ratio = 4.4) (Figure 1). In multivariate analyses, combined low TBS and BMD (odds ratio = 4.8, 95%CI: 1.02–22.9, *p*=0.04) and a presence of endocrinopathies (odds ratio = 4.4, 95%CI: 1.02–19.2, *p*=0.04) remained significantly associated with vertebral fractures (Table 3).

## 4. Discussion

The prevalence of thalassemia-associated osteoporotic fractures in beta-thalassemia major and in the various thalassemia syndromes has not been well established This study demonstrated that the prevalence of vertebral fracture was 16.3% (14/86), which was comparable with a previous study by Engkakul et al., which reported that the prevalence of vertebral fracture was 13% in Thai patients with thalassemia [26] The prevalence of vertebral fractures in the present study, however, was slightly higher than that in a previous report from a large cohort study including 31 clinical centers in the United States, Canada, and the United Kingdom (16.3% vs. 10.6%) [21]. The discrepancy in prevalence among the studies might be explained by three main factors including (i) the differences in the diagnostic methods of vertebral fracture because in the previous study, fractures were assessed by the self-reported fracture history questionnaires, but in this cohort, vertebral fractures included both symptomatic and/or radiographic vertebral fractures; (ii) the enrolled subjects in this study were adult patients with a high iron burden, and about 20% of patients had iron-associated endocrinopathies. Advanced age, iron overload, and endocrine disorders were important risk factors for osteoporosis and fractures in this population; and (iii) the ethnic differences in fracture risk [27].

Vertebral body bones are composed of the cancellous bone more than the cortical bone. The previous study demonstrated that low bone density, reduced trabecular bone volume, and extensive iron deposition were predominant characteristics of bone abnormalities in thalassemia bone disease [28]. These findings supported that the vertebral fracture in patients with thalassemia was more likely to be caused by thalassemia disease. Indeed, the TBS represents that the bone strength may be useful for assessing the risk of vertebral fractures. The association between low TBS and vertebral fractures, however, was demonstrated in postmenopausal women and men with the chronic obstructive pulmonary disease, and there was no study in thalassemia patients [29–31].

It is well established that a low BMD is an important risk factor for fractures in patients with thalassemia; however, this study showed that a low TBS was also associated with fractures. The current study found that patients with low TBS had a higher risk of vertebral fractures compared with low BMD (OR 9.2 vs. 4.4, *p* value <0.008 vs. 0.02). Moreover, patients with a combined low BMD and TBS were statistically significantly associated with vertebral fractures (OR 41.4, *p* value = 0.007). These findings suggested that thalassemia bone disease might explain the defect of both bone quantity and bone quality; however, impairment of bone quality might be more of a contributing factor for developing fractures in these patients than the bone quantity. The findings from this cohort suggested that all thalassemia patients should be evaluated for the TBS together with BMD for predicting the risk of fractures. T-L X-ray radiography should be performed in high-risk patients who have a combined low BMD and low TBS to exclude vertebral fractures.

Previous studies reported that the endocrine disorders were significant contributing factors of osteoporosis and fractures in patients with thalassemia. Endocrinopathies in these patients, for example, hypothyroidism, diabetes mellitus, hypogonadism, and hypoparathyroidism, led to increased osteoclast activity and decreased osteoblasts resulting in osteoporosis [7, 11, 13–15]. Fung et al. showed that hypogonadism was associated with fractures in patients with thalassemia [23]. Moreover, male patients with endocrine disorders, especially hypogonadism and a history of previous fractures, were predictive factors for future fractures [21].

An interesting finding in this present cohort was that pathological fractures were found in patients with *β*-thalassemia/Hb *E* and there were no fractures in patients with *a*-thalassemia. This finding might be explained by the nature of the disease that patients with *β*-thalassemia have more severe ineffective erythropoiesis compared to patients with *a*-thalassemia. A majority of patients with *β*-thalassemia/Hb *E* in this study were transfusion-dependent thalassemia. Supporting this, a large cohort study showed that fractures were more prevalent in patients with TDT and increased with age [20, 22]. Menopause, age, smoking, iron overload, and TDT, however, were not significantly associated with vertebral fracture in this study due to the small number of fracture events.

A limitation of this study is that the small sample size of patients limits the power to demonstrate the statistical significance of the potential clinical risk factors for fractures. The receiver operating characteristic (ROC) curve was constructed, but the sample size and the number of vertebral fractures were small to find the optimal cut-off point for predicting vertebral fracture in these patients. A large longitudinal cohort study is needed to confirm the associations of other clinical risk factors and fractures. To the authors' knowledge, however, this was the first study that demonstrated the association between radiographic vertebral fractures and the TBS in patients with thalassemia.

In conclusion, a combined low BMD and TBS and a presence of endocrinopathies were significant risk factors for vertebral fractures in patients with thalassemia. These findings could have clinical implications for performing BMD and TBS measurements as screening tools to evaluate the risk of vertebral fractures in patients with thalassemia, particularly in those patients with *ß*-thalassemia who have iron overload and endocrinopathies.

## Figures and Tables

**Table 1:** Baseline characteristics of 86 patients with thalassemia.

Characteristics	Transfusion-dependent thalassemia (TDT) (*n* = 57)	Non-transfusion-dependent thalassemia (NTDT) (*n* = 29)
Mean age (min-max), years	30.5 (18–60)	35.5 (18–58)
Mean pretransfused Hb (min-max), g/dL	7.2 (4.9–8.8)	7.6 (7.5–9.2)
Mean serum ferritin (min-max), ng/mL	2.690 (153–11,810)	1.651 (336–7,869)
Z-score BMD (min-max), g/cm^2^	0.7 (0.4–1.0)	0.8 (0.5–1.0)
TBS score (min-max)	1.2 (0.8–1.5)	1.3 (0.8–1.4)
Gender, *n* (%)		
Female	38 (66.6)	14 (48.3)
Male	19 (33.4)	15 (51.7)
Splenectomy, *n* (%)		
No	22 (38.6)	25 (86.2)
Yes	35 (61.4)	4 (13.8)
Vertebral fractures, *n* (%)		
No	48 (84.2)	26 (89.6)
Yes	11 (15.8)	3 (10.4)
Low lumbar spine BMD, *n* (%)		
No	30 (52.6)	20 (68.9)
Yes	27 (47.4)	9 (31.1)
Low TBS, *n* (%)		
No	53(93)	26 (89.6)
Yes	4 (7)	3 (10.4)
Smoking, *n* (%)		
No	51 (89.4)	23 (79.3)
Yes	6 (10.6)	6 (20.7)
Menopause, *n* (%)		
No	46 (80.7)	25 (86.2)
Yes	11 (19.3)	4 (13.8)
Endocrinopathies, *n* (%)		
No	44 (77.2)	24 (82.7)
Yes	13 (22.8)	5 (17.3)
Phenotype group, *n* (%)		
*β*-thalassemia/Hb E	52 (91.2)	15 (51.7)
Homozygous *β*-thalassemia	3 (5.2)	0 (0)
Hb H disease	0 (0)	5 (17.2)
Hb H disease with Hb CS	0 (0)	5 (17.2)
EABart's disease^*∗*^	2 (3.6)	4 (13.9)

Hb CS = hemoglobin constant spring; ^*∗*^compound heterozygous Hb H and heterozygous Hb *E*.

**Table 2:** Univariate analysis of risk factors for vertebral fractures.

Variables	OR	95% CI	*p* value
Age	1.02	0.9–1.1	0.46
Female gender	0.8	0.2–2.5	0.7
Low TBS score	9.2	1.7–47	0.008
Low BMD	4.4	1.2–15.5	0.02
Combined low BMD and TBS	14	2.2–86.5	0.005
Smoking	3.2	0.8–12.6	0.09
Splenectomy	5.7	1.5–22.5	0.01
Hemoglobin <7 g/dl	0.8	0.2–2.8	0.7
Serum ferritin >1,000 ng/ml	1.0	0.3–3.6	0.9
Transfusion-dependent thalassemia	2.1	0.5–8.1	0.3
Menopause	3.4	0.9–12.4	0.06
Endocrinopathies	5.3	1.5–18.3	0.007

OR = odds ratio; 95% CI = 95% confidence interval.

**Table 3:** Multivariate analysis of risk factors for vertebral fractures.

Variables	OR	95% CI	*p* value
Age	1.0	0.9–1.1	0.2
Endocrinopathies	4.4	1.02–19.2	0.04
Combined low BMD and TBS	4.8	1.02–22.9	0.04
Smoking	3.2	0.6–17.4	0.1
Splenectomy	3.4	0.6–18.5	0.1
Serum ferritin > 1,000 ng/ml	1.1	0.2–5.7	0.8

OR = odds ratio; 95% CI = 95% confidence interval.

## Data Availability

The data that support the findings of this study are available upon request to the corresponding author.
